# Neuroprotective effect of indomethacin in normal perfusion pressure breakthrough phenomenon

**DOI:** 10.1038/s41598-020-72461-1

**Published:** 2020-09-22

**Authors:** Manuel Revuelta, Alvaro Zamarrón, Jose Fortes, Gregorio Rodríguez-Boto, Raquel Gutiérrez-González

**Affiliations:** 1 Department of Neurosurgery, Puerta de Hierro University Hospital, Manuel de Falla 1, Majadahonda, 28222 Madrid, Spain; 2 Department of Neurosurgery, La Paz University Hospital, Pº Castellana 261, 28046 Madrid, Spain; 3 Health Research Institute-Fundación Jiménez Díaz (IIS-FJD), Avda Reyes Católicos S/N, 28040 Madrid, Spain; 4 Department of Surgery, Faculty of Medicine, Autonomous University of Madrid, Arzobispo Morcillo 4, 28029 Madrid, Spain

**Keywords:** Medical research, Neurology

## Abstract

Loss of cerebral autoregulation in normal perfusion pressure breakthrough (NPPB) phenomenon has been reported in other Central Nervous System diseases such as neonatal intraventricular haemorrhage. Several studies have demonstrated that low-dose indomethacin prevents this latter condition. A previous rat model was used to resemble NPPB phenomenon. Study animals were distributed in 4 groups that received 3 doses of indomethacin at different concentrations prior to fistula occlusion 60 days after its creation. Control animals received saline solution. Intracranial pressure (ICP) increased in all groups following fistula creation, whereas mean arterial pressure (MAP) and cerebral perfusion pressure (CPP) decreased as a manifestation of cerebral hypoperfusion and intracranial hypertension. The administration of indomethacin was associated with raised MAP and CPP, as well as decreased ICP. Sodium fluorescein extravasation was slight in study animals when comparing with control ones. Histological analysis evidenced diffuse ischaemic changes with signs of neuronal apoptosis in all brain layers in control animals. These findings were only focal and slight in study animals. The results suggest the usefulness of indomethacin to revert, at least partially, the haemodynamic effects of NPPB phenomenon in this experimental model, as well as to reduce BBB disruption and histological ischemia observed in absence of indomethacin.

## Introduction

Normal perfusion pressure breakthrough (NPPB) phenomenon is a theoretical explanation that tries to clarify the clinical observation of the development of massive brain swelling and/or diffuse cortical brain haemorrhages following intracranial arteriovenous malformation (AVM) occlusion^1,2^. This comorbidity is usually observed during the ending stages of the closure procedure and/or the early postoperative period, in absence of any other cause that may explain the phenomenon^1–5^. The incidence has been estimated at 3–6% of all treated cases^3–5^.

The theory was first proposed by Spetzler et al.^1^, who considered that the cerebral hemisphere harbouring an AVM, particularly large-size ones, suffers hypoperfusion and ischemia as a result of a “vascular steal” phenomenon that the AVM exerts on cerebral blood flow (CBF). As this phenomenon becomes chronic, the adaptive ability of vascular autoregulation of normal brain tissue surrounding the AVM would be affected and reflected on the permanent arterial dilation in the brain tissue marginal to the AVM, a condition that attempts to increase arterial contribution to counteract vascular steal and, therefore, chronic hypoperfusion^1,6,7^. Moreover, structural changes in small capillaries, such as the absence of astrocytic foot process layer, would also appear as a consequence of chronic hypoperfusion^8^. However, contralateral hemisphere changes have been observed both in clinical and experimental studies^2^.

Disorders in cerebral vascular autoregulation property and its adaptive ability (both in capillaries and abnormal vessels) have also been described in other diseases that affect the Central Nervous System, such as intraventricular haemorrhage (IVH) in the preterm newborn. The pathophysiology of IVH in premature infants is considered multifactorial: the intrinsic fragility of the germinal matrix, the structural changes in the basal lamina of brain vasculature and the disturbance in maturation of astrocytes-endfeet or podocytes (which develop a double function: they are an element of the blood brain barrier (BBB) as well as a structural support of vessels)^9,10^. When IVH reaches enough size, a dehiscence in the ventricular ependyma appears and so haemorrhage extends and becomes periventricular–intraventricular^11^.

Cerebral vessels are affected in those two diseases due to immaturity during development or to the loss of the adaptive autoregulation. As a result, vessels are unable to adequately respond to sudden changes in the systemic arterial pressure, a fact that also affects intracranial pressure (ICP) and, therefore, cerebral perfusion pressure (CPP). These mechanisms have been studied and proposed as the most probable primary physiopathogenic ones in the development of NPPB phenomenon (what involves risk of secondary oedema and haemorrhage) or IVH in the treatment of intracranial AVMs or preterm newborn respectively. In this context, several studies have demonstrated low-dose indomethacin to be useful to prevent severe grades of IVH in extremely low birth weight premature newborns^10,12,13^.

Considering the physiopathogenic similarities described between NPPB phenomenon and preterm IVH, it is hypothesized the effect of indomethacin on the prevention of the former taking into account the results obtained in the prevention of the latter. Thus, the aim of the study is, in the first place, to assess the neuroprotective effect of indomethacin in the prevention of primary physiopathogenic mechanisms involved in the development of NPPB phenomenon following intracranial AVM occlusion and then if such effect could depend on the drug dose.

## Methods

The research was based on a previous experimental model^14^. The study was approved by the Animal Care and Use Committee (CEBA) of “Puerta de Hierro” University Hospital (reference CEBA 016/2012; date April 16th 2012). The care and handling of the animals were performed in accordance with the European Directive and National Regulations and Guidelines for Animal Research. All invasive procedures (except drug administration) were performed under general anaesthesia (intramuscular injection of ketamine 75 mg/kg + xylazine 12 mg/kg, with rescue dose of 1/3 when needed in order to achieve adequate anaesthetic depth). Thirty male Wistar rats, 250–350 g in weight, were distributed in 5 groups of 6 animals each. All animals underwent a surgical procedure under general anaesthesia on day 0 which was divided in two consecutive stages: a *minor*-initial one and a *major*-final one. *Minor* procedure involved the placement of a sensor for ICP registration in the left-brain hemisphere by means of a post-coronal trepanation and durotomy. *Major* procedure entailed the creation of a left arteriovenous fistula (AVF) by means of an end-to-side anastomosis between the external jugular vein and the common carotid artery, similar to the one described in the previous model^14^. Bilateral external carotid arteries were also ligated.

**Figure 1:**
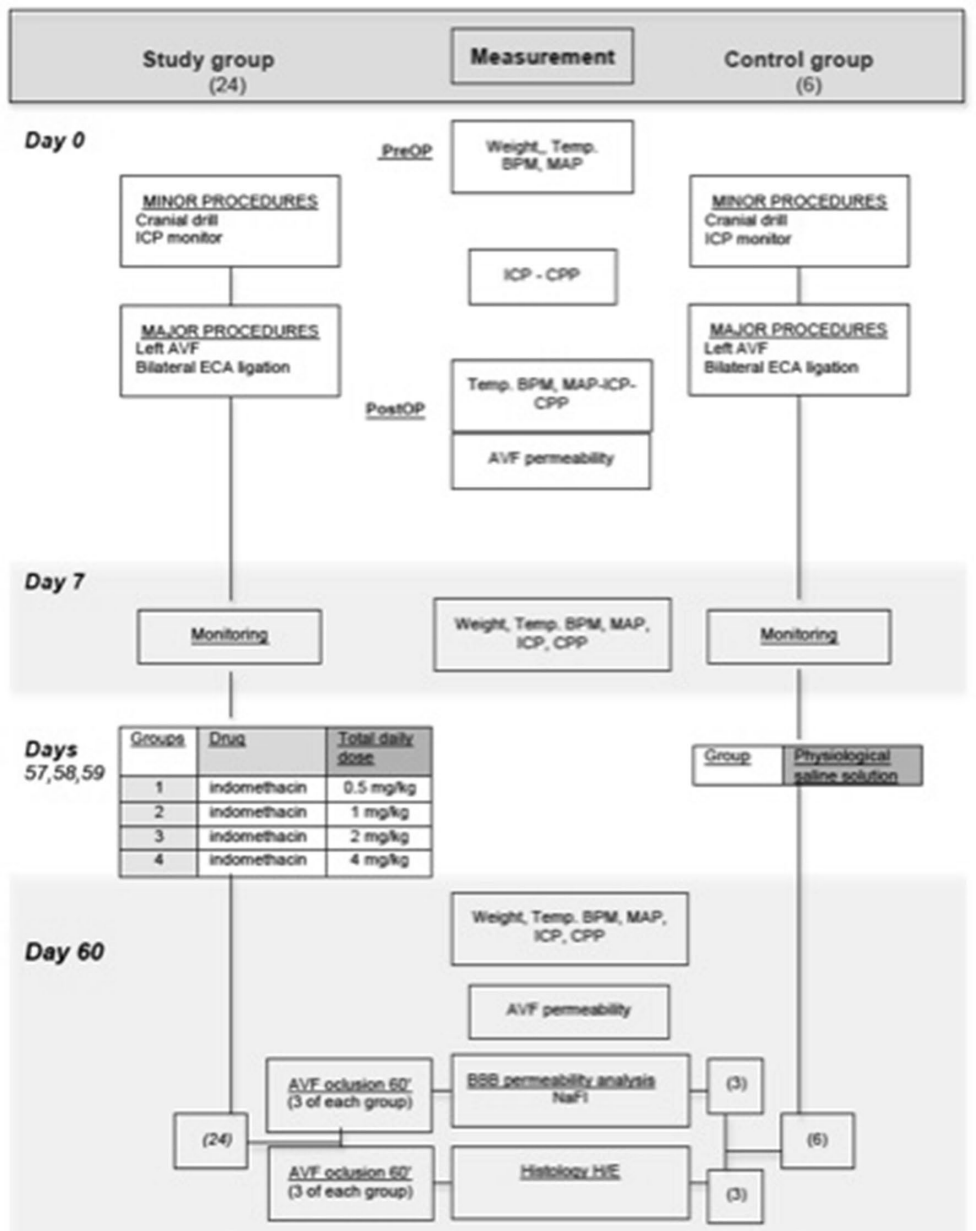
Flow chart of surgical procedures and variable measurements in study and control groups during the experiment.

Baseline (weight, temperature and heart rate) and haemodynamic variables (ICP, mean arterial pressure (MAP) and CPP) were recorded at the different stages of the experiment (Fig. 1). A non-invasive blood pressure measuring device (Harvard Apparatus, PanLab) and an intracranial catheter (Camino Intraparanchymal Sensor, Integra) were used for this purpose. Both MAP and ICP were registered for 5 min (in 6 different measurements) and mean values were calculated. Cerebral perfusion pressure resulted from the difference between MAP and ICP. All parameters were compared in study and control groups.

Anastomosis permeability was also checked with a doppler ultrasound device (Mizuho 20 Hz Doppler system; Mizuho Medical Corporation) postoperatively and on day 60 of the experiment.

A single daily dose of indomethacin was administered in the *study group* by subcutaneous injection in the left thigh of the rat. Up to three doses were administered in each animal; the first dose was administered on day 57 of the experiment and the last dose was administered 24 h prior to euthanasia. The study group was divided in 4 subgroups (each of which consisted of 6 animals) depending on the drug dose administered. Categorization of subgroups was named in order according to ascending dose administered. Total daily dose was calculated in accordance with each animal’s weight (Table 1).

**Table 1:** Experiment groups.

Groups	Drug	Total daily dose
1	Indomethacin	0.5 mg/kg
2	Indomethacin	1 mg/kg
3	Indomethacin	2 mg/kg
4	Indomethacin	4 mg/kg
Control	0.9% saline solution	0.5 mL

*Control group* (formed by 6 animals) was also inoculated by subcutaneous injection in the left thigh of each rat. A comparable volume of 0.9% saline solution was administered instead of indomethacin, following the same pattern. A total of three doses were provided in each animal; the first dose was administered on day 57 and the last dose was administered on day 59 of the experiment.

At the end of the experiment (day 60) and under general anaesthesia AVF was ligated during 60 min in all animals in order to resemble NPPB phenomenon^14^. Thereafter, and with the aim of evaluating BBB integrity, three animals of each group were perfused with fluorescein sodium (10% NaFl—0.6 mL/kg) via transcarotid injection. A thoracotomy was then performed and perfusion with saline solution was achieved until complete clearance in the right ventricle was confirmed. Euthanasia of all animals was held by decapitation and brain samples were collected for analysis. Staining pattern was documented by digital photography.

At this same point, and after AVF closure for 60 min, the remaining three animals in each group underwent transcardial perfusion with 4% paraformaldehyde solution in phosphate buffered and were euthanized following a previous model^14^. Brain samples were collected and fixed in formalin. Subsequently they were paraffin-embedded for haematoxylin and eosin stain evaluation. An independent observer analysed the samples and described pathological findings.

The neuroprotective effect of indomethacin was evaluated through hemodynamic response, BBB integrity and histological assessment.

Quantitative data collection was prospectively accomplished in an individual registration form, which was transferred to a database for analysis. Figure 1 describes the flow chart of the invasive procedures and measurements performed in all groups during the experiment. Statistical analysis was accomplished by Stata v 15.1 (StataCorp. 2017. Stata Statistical Software: Release 15. College Station, TX: StataCorp LLC).

Variables distribution followed an asymmetrical function. A descriptive analysis was performed using central tendency (median) and dispersion parameters (range; interquartile range). All parameters were compared between each of the different study subgroups and the control group. Considered level of significance was 5%. The evolution of different variables along the time was also evaluated. Since each animal was evaluated several times during the experiment, regression models were used in order to consider the correlation between data in a same animal. For this purpose, coefficients were obtained for each variable by means of mixed models (random effect). Besides that, regression coefficients, lineal predictions and their respective 95% confidence intervals were obtained to provide a better understanding of the different considered parameters evolution.

### Ethics approval

The study was approved by the Animal Care and Use Committee (CEBA) of “Puerta de Hierro” University Hospital (reference CEBA 016/2012; date April 16th 2012).

## Results

A total of 39 animals were needed to achieve the experiment. Eight of them presented early mortality (first 48 h of the experiment) and 1 presented delayed mortality, so 30 animals where considered for final analysis. In addition to fistula permeability corroboration by means of cervical Doppler ultrasound, surgical exploration on day 60 evidenced fistula patency in all cases, showing varicose, dilated and tortuous external jugular veins as well as hypoplastic common carotid arteries distal to the anastomosis. Retrograde flow from the carotid artery to the anastomosed jugular vein was also confirmed.

At the beginning of the experiment, all groups were homogeneous regarding control group when comparing weight and temperature. Heart rate was significantly higher in control group and MAP was only significantly different in study group 2. All groups were also homogeneous when considering ICP and CPP (Tables 2, 3).

**Table 2:** Baseline and hemodynamic variables in all experiment groups.

	Group 1 (0.5 mg/kg)	Group 2 (1 mg/kg)	Group 3 (2 mg/kg)	Group 4 (4 mg/kg)	Control group
**Day 0, preop**					
Weight (g)	315 (305–330)	315 (310–320)	307.5 (300–315)	317.5 (295–335)	297.5 (290–315)
Tª (ºC)	34.55 (34.2–35.9)	35.5 (34.9–35.9)	34.55 (34–35.2)	34.9 (34.8–35.9)	35.65 (34.8–36.2)
BPM	344 (321–357)	365.5 (317–428)	390.5 (357–417)	362 (342–375)	450 (403–465)
MAP (mmHg)	112.5 (107–117)	104 (97–107)	118 (107–129)	124.5 (115–127)	126 (122–127)
ICP (mmHg)	9.5 (6–11)	7 (5–8)	9 (8–9)	6.5 (6–11)	9.5 (7–11)
CPP (mmHg)	104 (95–109)	95.5 (90–101)	109 (98–121)	114.5 (108–118)	114.5 (114–117)
**Day 0, postop**					
Tª (ºC)	33.05 (32.7–33.1)	33.45 (33.1–33.8)	33.85 (33.8–34)	34.2 (33.4–34.4)	33.35 (32.8–33.8)
BPM	386.5 (360–403)	381 (340–428)	386 (338–414)	387.5 (372–390)	407 (385–428)
MAP (mmHg)	91.5 (84–101)	88.5 (80–93)	94 (92–101)	102.5 (90–110)	97 (92–99)
ICP (mmHg)	11 (10–14)	11 (7–12)	13 (11–14)	9.5 (9–14)	13.5 (12–16)
CPP (mmHg)	81 (69–87)	79 (68–84)	82.5 (78–91)	90.5 (76–101)	83 (79–83)
**Day 7**					
Peso (g)	262.5 (255–285)	230 (230–260)	282.5 (250–295)	247.5 (205–310)	262.5 (255–270)
Tª (ºC)	33.9 (32.7–34.5)	33.75 (32.7–34.2)	34.2 (33.6–35.8)	34.2 (33.9–34.7)	34.15 (33.7–35.6)
BPM	374 (367–424)	367 (342–415)	351 (323–389)	396.5 (366–411)	374 (324–399)
MAP (mmHg)	91 (76–96)	82 (81–87)	88 (83–99)	93.5 (82–111)	97.5 (92–106)
ICP (mmHg)	13.5 (11–14)	11 (9–14)	12.5 (12–14)	12 (10–14)	12 (9–15)
CPP (mmHg)	78.5 (62–83)	69 (67–75)	75.5 (67–85)	82 (68–103)	89 (80–92)
**Day 60**					
Peso (g)	382.5 (350–400)	327.5 (270–375)	342.5 (300–345)	277.5 (215–365)	340 (255–400)
Tª (ºC)	34.25 (34–34.6)	35.2 (34.7–36.2)	35.2 (33.9–36)	34.95 (34.7–35.1)	35.1 (34–35.9)
BPM	362 (298–377)	348 (325–371)	387 (346–424)	373.5 (348–424)	372 (335–415)
MAP (mmHg)	107 (95–108)	96.5 (90–100)	104.5 (98–119)	110.5 (106–123)	98.5 (97–109)
ICP (mmHg)	11.5 (8–13)	9.5 (8–12)	10 (10–10)	7.5 (7–13)	14.5 (11–17)
CPP (mmHg)	95.5 (82–100)	87 (80–89)	94.5 (89–109)	100.5 (99–110)	86 (80–92)

Values expressed as median (IQR).

**Table 3:** Comparison of haemodynamic variables between each study group and control group (*p* value).

	Group 1 (0.5 mg/kg)	Group 2 (1 mg/kg)	Group 3 (2 mg/kg)	Group 4 (4 mg/kg)
**Day 0, preop**				
BPM	**0.002**	**0.028**	0.182	**0.011**
MAP (mmHg)	0.077	**0.006**	0.573	0.838
ICP (mmHg)	0.965	0.230	0.829	0.081
CPP (mmHg)	0.590	0.072	0.613	0.960
**Day 0, postop**				
BPM	0.097	0.335	0.756	0.189
MAP (mmHg)	0.101	**0.044**	0.290	0.223
ICP (mmHg)	0.446	0.446	0.799	0.374
CPP (mmHg)	0.087	**0.039**	0.291	0.183
**Day 7**				
BPM	0.096	0.07	0.449	0.098
MAP (mmHg)	0.491	0.596	0.634	0.958
ICP (mmHg)	0.075	0.057	0.099	0.127
CPP (mmHg)	0.763	0.9	0.436	0.725
**Day 60**				
BPM	0.118	0.252	0.264	0.16
MAP (mmHg)	**0.009**	**0.005**	0.064	**0.016**
ICP (mmHg)	0.052	0.253	**0.005**	**0.011**
CPP (mmHg)	**0.004**	**0.004**	**0.021**	**0.005**

Bold values when p < 0.05.

All animals (study and control) showed a similar pattern regarding weight during the research development. According to the previous model^14^, weight significantly decreased at an early stage, with a subsequent recovery up to basal situation on day 60 (Table 2).

Temperature ranged from 32.7 to 36.5 °C during the development of the experiment. Overall median was 34.8 °C. The behaviour of this variable was homogeneous in all groups.

An overall slightly upward trend was observed in study groups regarding heart rate following AVF creation, in absence of any statistically significant differences when comparing with control group (Tables 2, 3). Stable values were registered on days 7 and 60 of the experiment among all groups, with an overall median value of 374 and 364 bpm, respectively.

### Haemodynamic measurement

#### Mean arterial pressure

Overall preoperative MAP value on day 0 ranged from 95 to 136 mmHg (median 116.5 mmHg). No difference was observed at this point among groups except in study group 2, which showed a significant reduced value regarding control animals. Postoperative value on day 0 registered a decline in median values in all groups when comparing with the preoperative status. The most significant decrease was recorded in study group 3 with a decrease of 24 mmHg, and the less significant decrease was recorded in study group 2, with a decrease of 15.5 mmHg. A slight decrease was also observed on day 7 in the study group. However, it was less intense than the one observed postoperatively. This trend was more pronounced in study group 4, where the median decreased in 9 mmHg comparing with the previous measurement. On the other hand, the value was stable in the control group (97.5 mmHg). Thus, no statistically significant difference was observed on MAP values on day 0 postoperatively and on day 7 when comparing groups, except for study group 2, which remained significantly decreased postoperatively (*p* = 0.044). Such difference was not present on day 7 (Tables 2, 3).

**Figure 2:**
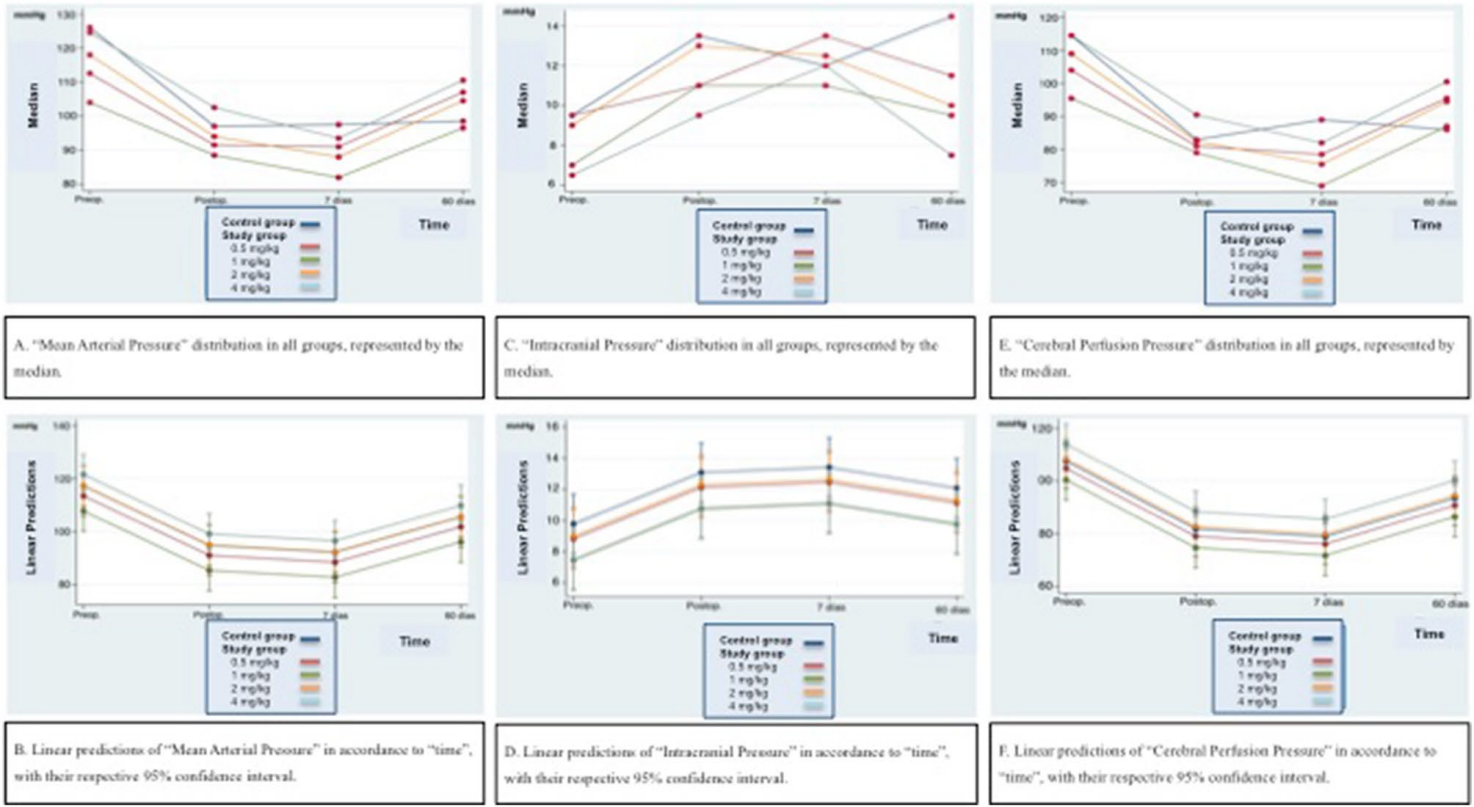
(**a**,**c**,**e**) Distribution of MAP, ICP and CPP in all groups, represented as the median. (**b**,**d**,**f**) Linear predictions of MAP, ICP and CPP in accordance to time with their respective 95% confidence interval.

A generalized increase in MAP was observed on day 60, after indomethacin administration, in the study groups. The most important increase was recorded in study group 4 (17 mmHg) and the less important one in study group 2 (14.5 mmHg). These findings were statistically significant when comparing study groups 1, 2 and 4 to control group (*p* = 0.009, 0.005 and 0.016, respectively). No difference could be related to indomethacin dose. Figure 2A,B resume MAP pattern during the experiment.

#### Intracranial pressure

Overall preoperative median value was 8.5 mmHg (range 4–13 mmHg). All study groups were homogeneous at the beginning of the experiment. Postoperative value increased in all groups. The most significant increase was recorded in the control group (4 mmHg with regard to the previous condition). The differences observed among groups were not statistically significant both preoperatively and postoperatively on day 0. Values were stable in all groups on day 7, with an overall median of 12.5 mmHg (range 5–17 mmHg). However, a mild upward trend was observed in study groups 1 and 4 with higher values comparing with the previous condition; on the other hand, a mild downward trend was recorded in study group 3 and control group. The differences observed were, again, not statistically significant (Tables 2, 3).

At the end of the experiment (day 60) prior to the fistula occlusion and after drug administration, widespread values in the different groups were registered (range 6–18 mmHg). However, values registered in the 4 study groups were significantly lower than those in the control group, except for study group 2 (*p* = 0.253). Again, no dose relationship was evidenced. Figure 2C,D resume ICP pattern along the experiment.

#### Cerebral perfusion pressure

Overall preoperative median value was 108.5 mmHg (range 88–128 mmHg). Higher values were observed in control and study 4 groups (114.5 mmHg); lower median corresponded to study group 2 (95.5 mmHg). All groups were homogenous at this stage. Postoperative value decreased in all groups, with an overall median of 83 mmHg (range 59–103 mmHg). However, this decline was only significant in study group 2 (*p* = 0.039). Values were stable in almost all groups on day 7, with an overall median of 77.5 mmHg. The differences observed among groups were not statistically significant (Tables 2, 3).

At the end of the experiment (day 60) and after indomethacin administration, all study groups showed a more pronounced increase in CPP when comparing with the control group. Theses differences reached statistical significance (Tables 2, 3) and were not dependent on indomethacin dose. Figure 2E,F resume CPP pattern during the experiment.

#### Blood brain barrier permeability assessment

**Figure 3:**
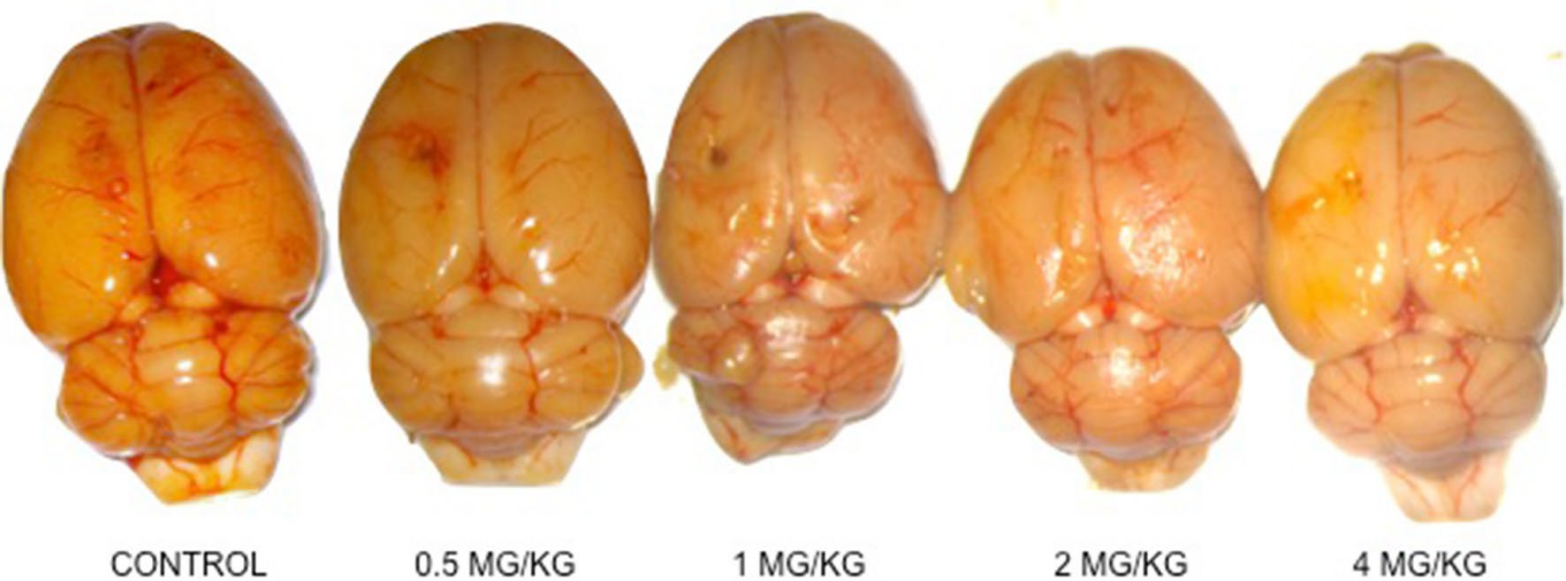
Rat brain photograph of control and study groups. Sodium fluorescein extravasation is evidenced. Staining is lighter in those specimens that had previously received indomethacin when comparing with control specimen.

NaFl extravasation was evidenced in all animals of control and study groups following the restoration of CPP after arteriovenous fistula closure. The staining distribution was homogeneous and affected both hemispheres. However, staining was lighter in those specimens that had previously received indomethacin when comparing with control specimens. No differences could be attributed to the drug dose administered in each case (Fig. 3).

#### Histological exam

**Figure 4:**
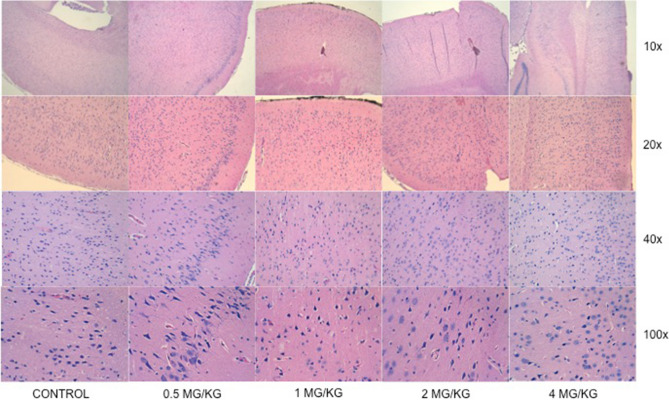
Light microscopy (haematoxylin and eosin staining): comparison between control and study groups (labelled by indomethacin dose received) at different augmentation.

Light microscopy evaluation (haematoxylin and eosin staining) did not evidence foci of oedema and/or haemorrhage. Both control and study specimens showed morphological changes in neurons related to cell necrosis (reduced cytoplasm size together with pyknotic neuronal nuclei). The extent of this finding was wide in the control group, with presence of such changes in all analysed layers of the brain cortex. On the contrary, the presence of these morphological changes was only localized and focal—patch-characterized—in the study group. No difference could be attributed to indomethacin dose (Fig. 4).

## Discussion

Several therapies have been proposed to prevent NPPB phenomenon, a complication that involves high morbidity and mortality. However, no therapy has been clearly demonstrated to prevent it up to date. A progressive increase in blood perfusion of the ischemic hemisphere was initially proposed. Gradual and staged obliteration of the AVM by means of sequential closure of feeding arteries was achieved^1,2^. Endovascular and/or surgical procedures (vascular clips or ligature) were used for this purpose^1,2,15,16^. Staged resection of the AVM has also been suggested^1,17^. However, new cases of this complication have been reported despite these existing measures^2^.

Systemic arterial hypotension has also been considered to prevent NPPB, particularly in the early postoperative period^1,18–20^. On the contrary, it is a controversial measure during the surgical procedure, since it may worsen pre-existing ischemia^21,22^. Other authors, however, defend it^15,17^. Local hypotension has also been attempted by means of a gradual and reversible closure of ipsilateral carotid artery in its cervical segment, and during a variable period of time. This procedure was described both intraoperatively (during AVM resection) and postoperatively, but no conclusive recommendation could be reached^23,24^.

Indomethacin has been previously considered in the treatment of raised ICP in patients with traumatic brain injury, due to a vasoconstrictor effect and a decrease in CBF^25^. Similarly, it has been used in the treatment of established NPPB phenomenon, but it was not recommended since early signs of brain ischemia were detected^26^. Other postoperative recommendations include intensive care unit surveillance during at least 24 h^17^, negative fluid balance (osmotherapy and diuretics)^17,20,23^, control of ICP^17,20,23^ and/or even barbiturate coma therapy when needed^20,23^.

Immunohistochemistry studies based on the detection of glial (glial fibrillary acidic protein—GFAP- and aquaporin 4) and vascular markers (laminin) have determined that podocyte density is similar in the whole brain tissue regardless of gestational age. However, podocytes differentiation at the germinal matrix is less numerous when compared with the rest of the encephalon (less concentrated GFPA in spite of similar aquaporin 4 with regard to any other location)^27^. This finding has also been considered a possible marker of fragility associated with potential risk of secondary haemorrhage^23^. Other studied factors involved in CBF regulation during brain development are cyclooxygenase enzymes expression and subsequent production of prostaglandin^28,29^. Furthermore, microglia reacts against hypoxia by releasing free radicals (which are known to damage the endothelium, alter haemostasis and increase anaerobic metabolism when they are not well cleared)^30,31^. Central Nervous System in extremely low birth weight premature newborns is more likely to suffer free radical damage due to clearance system immaturity^10^. A significant increase in CBF occurs as a result of the following variations: sudden arterial pressure changes (mainly hypotension)^32^; states of arterial-blood gas concentration (hypoxemia e hypercapnia)^28^; or changes in blood pH (acidosis)^28,32,33^. This fact initially favours haemorrhage in the germinal matrix but may subsequently extend to the ventricular system^10^.

Indomethacin is widely accepted in the preterm newborn to prevent patent ductus arteriosus^34^. It has also been proven to prevent severe grades of IVH in the preterm newborn^10,12,13^. Thus, both incidence and severity of IVH may be reduced with indomethacin administration, according to experimental and clinical studies^12,13^. However, long-term benefits are controversial^12,13^. Prevention of IVH would be achieved by means of a double mechanism. On one hand, by the effect on CBF; on the other hand, by promoting basal membrane maturation^28,32,33,35,36^. Low-dose administration of indomethacin in extremely low birth weight premature newborns has been demonstrated to associate neuroprotective characteristics such as: the reduction of sudden increases in CBF temporarily in cases of hypoxia/hypercapnia, with subsequent recovery of basal values to widen the range where CBF autoregulation acts, specially in cases of increased CPP such as severe hypoxia in preterm newborn^13,28,32,37–39^; to the increase in cerebral vascular resistance in absence of changes in heart function^37^; the achievement of structural maturation of vessels involved in periventricular germinal matrix by means of laminin and collagen store^36^; the reduction of BBB permeability^40^; the prevention of free radicals formation such as superoxide anion, (which is well known to contribute in reperfusion damage)^41^.

Changes related to cerebral perfusion disorder, such as hypoxia, hypercapnia and arterial hypotension usually occur following labour of extremely low birth weight premature newborns^13,42^. Thus, IVH in these infants has been attributed to changes in CBF in the periventricular germinal matrix, due to the particular structure of the matrix and micro vessels immaturity^10^. Indomethacin administration during the first day of life has been proven as an effective measure to prevent IVH, particularly severe grades^12,42–44^. Thus, indomethacin may reduce the hyperemic response favouring cerebral autoregulation^28,32,33^, promoting microvascular maturation in the germinal matrix^32,36,41^, and inhibiting changes in BBB permeability^28^.

In the model hereby described indomethacin administration pattern was discussed and decided based on its effect on intraventricular haemorrhage in the preterm newborn. It was administered prior to AVF occlusion since we were looking for a preventive effect, rather than a therapeutic effect. Extending indomethacin administration after AVF closure would imply more interventional procedures that would need anaesthesia administration in the rat and may increase mortality rate^14^. However, we do not discard to test it in further investigations.

The present study represents the first one that analyses the usefulness of indomethacin in the prevention of NPPB phenomenon in an experimental animal model that resemble primary physiopathogenic phenomena involved in it. Prophylactic and therapeutic measures employed in NPPB phenomenon do not have evidence enough to be widespread recommended up to date^1,2,16,17,23,24,45^. Indomethacin administration prior to fistula occlusion reduced ICP and improved CPP. Sodium fluorescein extravasation was used for a qualitative assessment of BBB damage. Staining distribution was bilateral and symmetrical in all specimens, which shows a not only a focal but global disorder. These findings are similar to previous observations^45–47^. However NaFl staining was macroscopically fainter in animals from all study groups rather than in animals from the control group in absence of dose-related differences. Neuronal degeneration and cellular necrosis have been previously described^48^. These findings were also attenuated when indomethacin was used in the experiment.

This study comprises also some limitations to be mentioned. Conclusions are based on an experimental model that although reproduces the primary physiopathogenic events involved in the subsequent development of NPPB phenomenon, it does not resemble the final and dreaded complications associated to it (oedema and/or haemorrhage). However, the opportunity to know and understand such physiopathogenic mechanisms is essential to consider indomethacin as a prophylactic alternative in the management of intracranial AVMs. This is a preliminary study that will be implemented with added measurements in further experiments, such as cortical cerebral blood flow (by a laser speckle flowmetry imaging system) or ICP pulse amplitude.

Sodium fluorescein extravasation entails a qualitative but not quantitative assessment of BBB integrity. A different method that allows a deeper and more exact knowledge of distribution patterns in NPPB phenomenon, such as polarized light, should be tested.

Despite light microscopy evidenced neuronal degeneration and pyknosis^48^, it was not able to show haemorrhage or apoptosis. Further studies with more specific techniques, such as electronic microscopy, flow cytometry, and TUNEL assay may help assess the effect of indomethacin in NPPB phenomenon more accurately.

## Conclusions

Indomethacin administration prior to AVF closure was related to a non dose-dependent significant increase in MAP and CPP. Besides that, ICP showed a drop after indomethacin administration that was significant in most of the study groups. Indomethacin employment also allowed to a partial weaken BBB disruption related to NPPB phenomenon as well as the restriction of neuronal degeneration and pyknosis, according to fluorescein staining and histological assessment, respectively.

Thus, the results obtained in this study show that the use of indomethacin prior to AVF closure was related with the partial prevention of primary mechanisms of NPPB phenomenon, mainly due to its effect on hemodynamic variables and, in a lesser extent, due to its effect on BBB disruption and neuronal degeneration. Further studies including more specific techniques and testing other indomethacin administration patterns are needed to confirm such observations.
